# Effects of Continuous Sugar Beet Cropping on Rhizospheric Microbial Communities

**DOI:** 10.3390/genes11010013

**Published:** 2019-12-22

**Authors:** Weijuan Huang, Donglei Sun, Jiantao Fu, Huanhuan Zhao, Ronghua Wang, Yuxing An

**Affiliations:** 1 Guangdong Bioengineering Institute (Guangzhou Sugarcane Industry Research Institute), Guangdong Province Pesticide-fertilizer Technology Research Center, Guangzhou 510316, China; huang@muc.edu.cn (W.H.);; 2 Shihezi Academy of Agricultural Sciences, Xinjiang Uygur Autonomous Region, Shihezi 832000, China

**Keywords:** sugar beet, continuous cropping, rhizosphere microbial community, high-throughput sequencing

## Abstract

The continuous cropping of sugar beet can result in soil degradation and a decrease in the sugar beet yield and quality. However, the role of continuous sugar beet (*Beta vulgaris* L. var. saccharifera) cropping in shaping the structure and function of the rhizosphere microbial community remains poorly investigated. In this study, we comparatively investigated the impact of different numbers of years of continuous sugar beet cropping on structural and functional changes in the microbial community of the rhizosphere using high-throughput sequencing and bioinformatics analysis. We collected rhizosphere soils from fields continuously cropped for one-year (T1), five-year (T5), and thirty-year (T30) periods, as well as one bulk soil (T0), in the Xinjiang Uygur Autonomous Region. The results demonstrated that continuous sugar beet cropping resulted in a significant decline in the community diversity of soil bacterial and fungal populations from T1 to T5. With continuous change in the structure of the microbial community, the Shannon diversity and observed species were increased in T30. With an abundance of pathogenic microbes, including *Acidobacteria*, *Alternaria*, and *Fusarium*, that were highly enriched in T30, soil-borne diseases could be accelerated, deduced by functional predictions based on 16S rRNA genes. Continuous sugar beet cropping also led to significant declines in beneficial bacteria, including *Actinobacteria*, *Pseudomonas* spp., and *Bacillus* spp. In addition, we profiled and analyzed predictive metabolic characteristics (metabolism and detoxification). The abundance of phenolic acid decarboxylase involved in the phenolic acid degradation pathway was significantly lower in groups T5 and T30 than that in T0 and T1, which could result in the phenolic compounds becoming excessive in long-term continuous cropping soil. Our results provide a deeper understanding of the rhizosphere soil microbial community’s response to continuous sugar beet cropping, which is important in evaluating the sustainability of this agricultural practice.

## 1. Introduction

Sugar beet (*Beta vulgaris* L. var. *saccharifera*) is an important crop in temperate climates, providing approximately 30% of the world’s annual sugar production [1]. Sugar beet is an indispensable and important sugar crop in the north and west of China and is the main crop cultivated in Heilongjiang, Inner Mongolia, and Xinjiang areas [2]. However, the production of sugar beet is strongly influenced by natural geographical conditions, and is currently grown using relatively backward farming methods and cultivation techniques [3]. Continuous cropping has aggravated more problems in sugar beet systems, including soil-quality degradation, crop-yield and sugar content reduction, and plant disease intensification [4].

Studies have found that continuous cropping systems are bottlenecks that restrict the sustainable development of agriculture. Three factors are thought to determine the problems associated with continuous cropping: an imbalance in soil nutrients, autotoxicity of root exudates, and shifts in the microbial community composition [5]. Continuous obstacles to the cropping of sugar beet could become severe due to the shortage of cultivated land resources in the Xinjiang area, as well as the imperfect cultivation management system and the constraints of the production environment. Comprehensive prevention and control technologies have been reported to be effective measures for alleviating such problems, including the use of improved seeds, new fertilizers, environmentally-friendly pesticides, and soil conditioners [6,7,8]. However, studies on the control of obstacles to continuously cropping sugar beet using comprehensive solutions and key agricultural technologies are still rare.

Rhizosphere microflora has been shown to be a key component of agricultural ecosystems that not only plays a significant role in basic soil processes, but is also actively involved in enhancing soil fertility and crop productivity [9]. Previous studies on soybean, ginger, potato, cucumber, and cotton have found that the alpha diversity of bacteria and fungi was decreased due to continuous cropping year, and the soil microbial metabolic diversity and soil physicochemical properties were also significantly affected [10,11,12,13]. The relative abundance of pathogenic fungi in rhizospheric soil was revealed to be significantly higher in continuous cropping than in cropping rotation systems [14]. However, the mechanism underlying the effect on soil microbial communities and the relationship between these effects and continuous cropping systems have not yet been fully elucidated for sugar beet. Here, we aimed to find out the diversity and structure of microbial communities of rhizosphere soil altered by 1-, 5-, and 30-year continuous sugar beet cropping using high-throughput sequencing techniques.

## 2. Materials and Methods 

### 2.1. Study Sites and Sample Collection

Twelve samples of rhizosphere soil were collected from three different years of continuous sugar beet cropping in Shihezi City, Xinjiang Uygur Autonomous Region, including 1-year (86°00′98″ N, 44°31′32″ E), 5-year (86°01′41″ N, 44°31′38″ E), and 30-year (86°01′59″ N, 44°31′25″ E) continuous cropping systems, as well as a rotation soil as a control (86°05′39″ N, 44°34′02″ E) (three soil replicates for each group). The collection site has an annual average temperature of about 3.6~8.5 °C. The sunshine duration is around 2800~3150 h, and the accumulated temperature over 10 °C is about 2941~3500 °C [15]. Each rhizosphere soil sample was combined soil collected from three sugar beet plants, as described before [16]. Briefly, the mature sugar beet was carefully removed by a spade, and the plant was shaken vigorously to separate any soil not tightly adhering to the roots. Roots with soil on the surface were shaved off into a 10 mL sterile tube, sterile 1 × PBS (phosphate-buffered saline) solution was added (Solarbio Life Sciences, Beijing, China), and the sample was then centrifuged at 8000× *g* for 10 min at 4 °C. The resulting pellet, defined as the sample containing the rhizosphere-enriched microbial communities, was frozen in liquid nitrogen and stored at −80 °C.

### 2.2. DNA Extraction, PCR Amplification, and Sequencing

Microbial DNA from soil samples was extracted using a Soil Microbe DNA Kit (QIAGEN, USA). The DNA concentration and purity were monitored with 1% agarose gels. According to the concentration, DNA was diluted to 1 ng/µL using sterile water. Distinct regions of bacterial 16S rRNA and fungal ITS (Internal Transcribed Spacer) genes were amplified using primers, including the 16S rRNA V4 gene (515F: GTGCCAGCMGCCGCGGTAA; 806R: GGACTACHVGGGTWTCTAAT) and ITS1-5F gene (ITS5-1737F: 5′-GGAAGTAAAAGTCGTAACAAGG-3′; ITS2-2043R: 5′-GCTGCGTTCTTCATCGATGC-3′), respectively [17]. All PCR reactions were carried out in 30 µL reactions with 15 µL of Phusion^®^ High-Fidelity PCR Master Mix (New England Biolabs, MA, USA), 0.2 µM of forward and reverse primers, and about 10 ng of template DNA. Thermal cycling consisted of initial denaturation at 98 °C for 1 min, followed by 30 cycles of denaturation at 98 °C for 10 s, annealing at 50 °C for 30 s, elongation at 72 °C for 30 s, and finally 72 °C for 5 min. Sequencing libraries were generated using Ion Plus Fragment Library Kit 48 rxns (Thermo Scientific, Waltham, MA, USA), following the manufacturer’s recommendations. The library quality was assessed on the Qubit@ 2.0 Fluorometer (Thermo Scientific, Waltham, MA, USA) and Agilent Bioanalyzer 2100 system. Lastly, the library was sequenced on an Ion S5TM XL platform, and 400 bp/600 bp single-end reads were generated. All the high-throughput sequencing was done by Novogene Technology Co, Ltd. (Beijing, China).

### 2.3. Data Analysis

Sequence analysis was performed by Uparse software (Uparse v7, http://drive5.com/uparse/) [18]. The sequencing data were merged and filtered to remove the merged reads with a length of <400 bp or the quality score of <Q30 for more than 1% of the bases. Sequences with ≥97% similarity were assigned to the same operational taxonomic units (OTUs). A representative sequence for each OTU was screened for further annotation. For each representative sequence, the Silva Database (https://www.arb-silva.de/) [19] and UNITE (v7.2) (https://unite.ut.ee/) [20] were used, based on the Mothur algorithm, to annotate taxonomic information. Analysis of alpha diversity and beta diversity of the normalized dataset was performed using the R language and the Vegan package (published by Philip Dixon, 2003). The overall microbiome dissimilarities among all samples were accessed using the weighted UniFrac distance matrices [21]. Principle coordination analysis (PCoA) was used to visualize the dissimilarities. Differentially abundant features were performed using Metastat analysis in R. Microbial diversity and the relative abundance of different microbial taxonomic levels were assessed for differences between different groups with one-way ANOVA, a *t*-test, or a Wilcox test. LDA (Linear Discriminant Analysis) effect size (LEfSe) analysis was performed online (https://huttenhower.sph.harvard.edu/galaxy) to find differentially abundant taxa (biomarkers) with *P*-values higher than 0.05 and LDA scores higher than 2. Phylogenetic Investigation of Communities by Reconstruction of Unobserved States (PICRUSt) and FunGuild were used to predict the metabolic function of the microbial flora [22]. All raw sequence data are accessible in the NCBI Sequence Read Archive (SRA) database, under the BioProject number PRJNA592845.

## 3. Results

### 3.1. Diversity Indices and Richness of Microbial Communities

For the entire sampling set, a total of 1,381,255 bacterial sequences (raw tags) with an average length of 253 bp, and 877,043 fungal sequences with an average length of 228 bp, were identified using Illumina MiSeq analysis (Dataset S1 & S2). As a result of chimeral filtration and quality control, 1,321,435 bacterial and 830,144 fungal high-quality sequences (clean tags) were obtained. Finally, a total of 1,134,502 bacterial and 699,667 fungal effective tags were found in all samples, accounting for 95.7% and 94.8% of the total quantified sequences, respectively (Datasets S1 and S2). All the sampling efforts tended to reach the saturation plateau in rarefaction analysis and were effective in covering the full extent of almost a majority of the bacterial and fungal diversity according to 97% sequence similarity in the rank abundance curve approach (Supplementary Figure S1).

**Figure 1:**
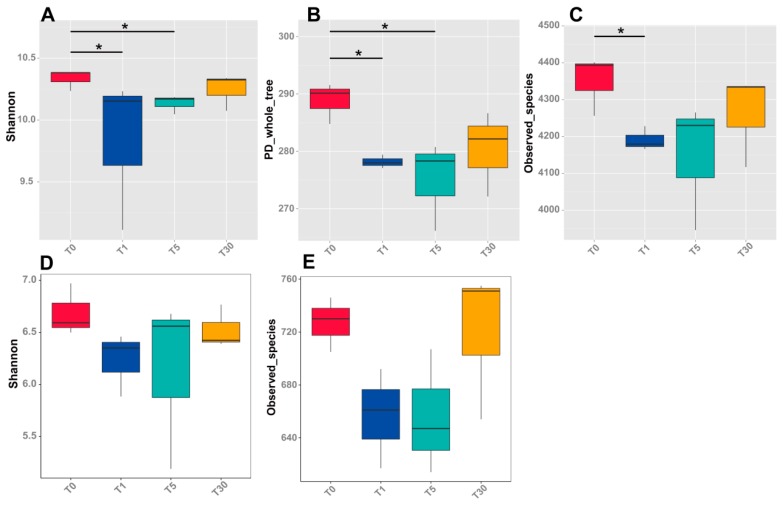
Alpha-diversity of the bacterial and fungal communities for four groups of microbial samples. (**A**–**C**), representing Shannon diversity, PD_whole tree, and observed species of the bacterial community, respectively; (**D**,**E**), representing Shannon diversity and observed species of the fungal community, respectively. Significances between different groups were compared using Wilcoxon’s test, with the results indicated on the top (*p*-value ≤ 0.05 = *).

The richness index, Shannon diversity index, and Observed Species indices are presented in Supplementary Tables S1 and S2 (Dataset S2 and S3), showing that the bacterial and fungal species number, richness diversity, and OTU ratio of bacteria/fungi decreased with an increasing number of years of continuous cropping. OTU numbers of bacteria decreased with consecutive sugar beet cropping from 4716 (T0) to 4504 (T30), but were not significantly different between T1, T5, and T30 (Supplementary Table S1). Shannon and PD_whole tree values of the bulk soil T0 were significantly higher than those of the 1-year (T1) and 5-year (T5) continuous cropping groups (Figure 1a,b, Table S1). The Observed Species index value of the bulk soil T0 was only significantly higher than that of T1 (Figure 1c). No significant differences were found between the 30-year continuous cropping group (T30) and the others. Fungal diversity indices, including Shannon and Observed Species indices, were higher in T0 and T30 than in T1 and T5, but not significantly (Figure 1d,e, Supplementary Table S2). 

**Figure 2:**
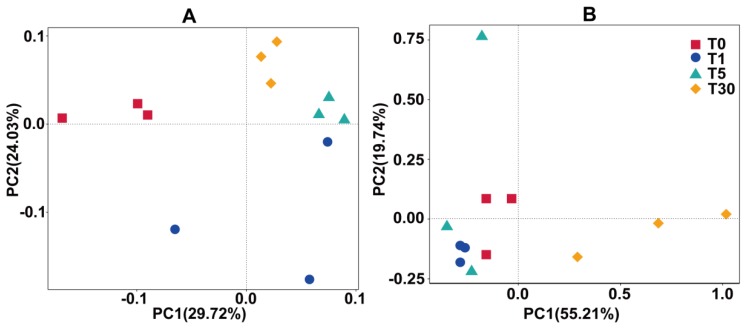
Beta diversity of the microbial community using principal coordinate analysis plots (PCoA) analysis with the weighted_Unifrac metric. (**A**) bacterial community; (**B**) fungal community.

According to the principal coordinate analysis plots (PCoA), the bacterial communities of each group were separated by PCo1 (29.72%) and PCo2 (24.03%) (Figure 2, Dataset S4). The PCoA analysis based on weighted UniFrac distances placed the T5 groups much closer to the T1 and T30 groups due to their similar bacterial community structure, while the bulk soil in T0 was distantly placed between three principal coordinates (Figure 2a). This demonstrated that the structure of the bacterial community after continuous cropping was affected more strongly than that of the bulk soil, which could be intensified by the root exudates or allelochemicals of sugar beet. The fungal communities of each group were separated by PCo1 (55.21%) and PCo2 (19.74%) (Figure 2b). The plot shows that the samples were separated into four groups, that is, the bulk soil (T0), one year of mono-cropping rhizospheric soil (T1), five years of mono-cropping rhizospheric soil (T5), and thirty years of mono-cropping rhizospheric soil (T30). This distribution pattern indicated that the fungal community structures in the T0 bulk soil and T1 and T5 rhizospheric soils were distinctly different from the T30 rhizospheric soil.

### 3.2. Microbial Community Composition and Structure Analysis

**Figure 3:**
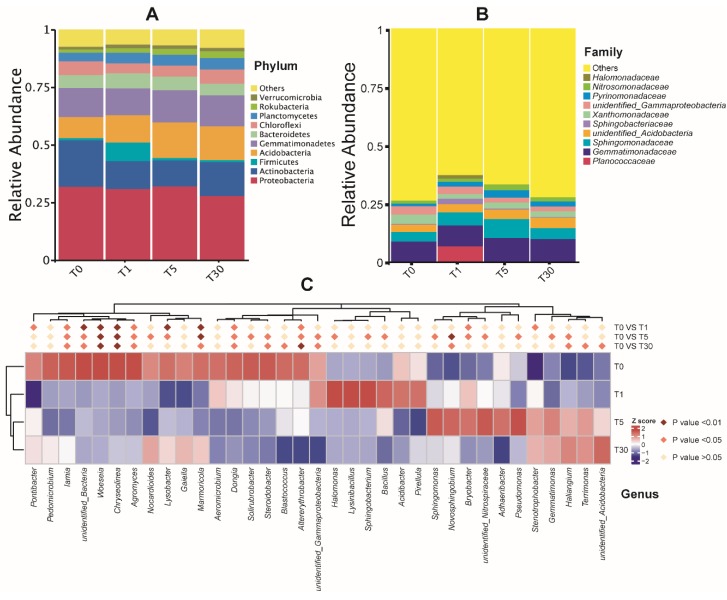
Taxonomic differences of the bacterial community among four groups of samples. (**A**) The top 10 phyla; (**B**) the top 10 families; (**C**) relative abundances of the top 20 genera were compared to T0 using MetaStat analysis.

The dominant bacterial phyla in different years of continuous cropping of sugar beet were Proteobacteria, Actinobacteria, Firmicutes, and Acidobacteria (Figure 3a, Dataset S5). At the family level, the bacterial communities in all groups were dominated by *Gemmatimonadaceae* (8%–10%) and *Sphingomonadaceae* (4%–8%), followed by *Xanthomonadaceae* (2%–4%), *Pyrinomonadaceae* (1%–3%), and *Nitrosomonadaceae* (1%–2.5%) (Figure 3b, Dataset S5). Interestingly, the abundance of *Planococcaceae* was found to be significantly increased in the T1 group (*P* < 0.05) compared with that in other groups. Meanwhile, the abundance of the unidentified_*Acidobacteria* family showed increases along with the year of sugar beet mono-cropping.

For the top 20 bacterial genera, the results showed that the sequences affiliated with the genera *Sphingomonas*, *Haliangium*, *Gaiella*, and *Lysobacter* successively increased, while *Lysinibacillus* and *Sphingobacterium* declined significantly with the increase in the number of years of continuous cropping (*P* < 0.05) (Figure 3c, Dataset S6). The abundance of *Halomonas*, *Sphingobacterium*, and *Bacillus* was significantly increased (*P* < 0.05) in T1 compared with that in T5. The relative abundance of *Pseudomonas*, *Sphingomonas*, *Novosphingobium*, and *Bryobacter* was significantly higher (*P* < 0.05) in T5 than in T0. *Haliangium*, *Terrimonas*, and *unidentified_Acidobacteria* were significantly increased (*P* < 0.05) in T30 compared to in T0. Further analysis showed that *Roseimicrobium* in both T1 and T5 was significantly higher (*P* < 0.01) than that in T30. Meanwhile, the abundance of *Gemincoccus* in T5 showed significant increases (*P* < 0.01) compared to that in T1.

**Figure 4:**
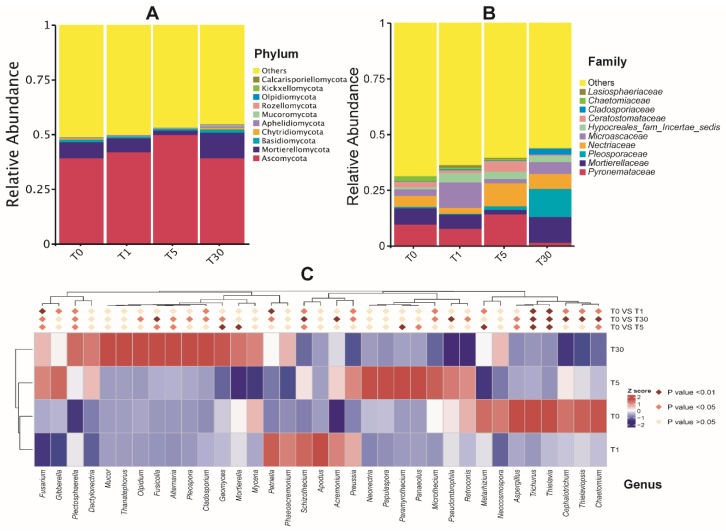
Taxonomic differences of the fungal community among four groups of samples. (**A**) The top 10 phyla; (**B**) the top 10 families; (**C**) relative abundances of the top 20 genera were compared to T0 using MetaStat analysis.

The dominant fungal phylum across all the samples was Ascomycota (ranging from 40% to 50%), followed by Mortierellomycota (2%–11.6%) and Basidiomycota (0.8%–1.5%) (Figure 4a, Dataset S5). Among the top ten phyla, Chytridiomycota, Aphelidiomycota, Mucoromycota, Rozellomycota, Olpidiomycota, Kickxellomycota, and Calcarisporiellomycota were minor phyla, with an average relative abundance of less than 1%. As shown in Figure 4b (Dataset S5), the relative abundance of the fungal family diverged significantly along with the duration of continuous sugar beet cropping. Surprisingly, Pyronemataceae, Nectriaceae, and Ceratostomataceae abundance increased from T1 to T5, and then decreased in T30. In contrast, Mortierellaceae and Microascaceae abundance decreased from T1 to T5, and then increased in T30. It is interesting to note that Pleosporaceae and Cladosporiaceae abundances were significantly increased in T30, suggesting that both of these fungi could be pathogenic and adapted to the soil environment after the long-term mono-cropping of sugar beet. In contrast, the abundance of Chaetomiaceae and Lasiosphaeriaceae significantly decreased from T0 to T30; these were proposed to be beneficial fungi that were severely affected by continuous cropping. For the top 20 fungal genera, the abundance of *Plectosphaerella*, *Cladosporium*, *Geomyces*, *Olpidum*, *Fusicolla*, *Pleospora*, and *Mortierella*, as well as the two plant pathogenic fungi *Fusarium* and *Alternaria*, was found to be significantly increased (*P* < 0.05) in T30 (Figure 4c, Dataset S7). Further statistical tests revealed that the genera *Selinia*, *Pseudallescheria*, and *Macroconia*, belonging to the dominant phylum Ascomycota, were significantly higher (*P* < 0.01) in T1, T0, and T5, respectively, compared with those in other groups (Supplementary Figure S2).

Based on an LEfSe analysis, we determined that the abundances of microbial species in the different groups were significantly different (Supplemental Figure S3a, Dataset S8). T5 was highly enriched in Acidobacteria, while T0 was highly enriched in Actinobacteria, indicating that beneficial microbes decreased with the increasing duration of sugar beet mono-cropping. Regarding fungi, the communities of different groups also varied, with the accumulation of pathogenic fungi caused by long-term sugar beet mono-cropping. The pathogenic fungus *Fusarium incarnatum* was highly enriched in T5, while *Alternaria alternata* was enriched in T30; both of these plant pathogens threaten the yield of sugar beets (Supplemental Figure S3b, Dataset S9). In addition, these four groups of different bacterial and fungal communities were clustered differently in the cladogram trees (Supplemental Figure S3). In particular, the abundances of the family Nectriaceae and the species *Fusarium incarnatum* in the T30 group were greatly separated from other groups in the tree.

### 3.3. Predictive Functions of the Rhizosphere Microbial Community

Bacterial functional profiles were inferred from 16S rRNA gene data using PICRUSt. We annotated 6092 bacterial OTUs in total (Dataset S10) to predict the metagenome function. Differences between the bulk soil and rhizosphere soils from different years of mono-cropping are shown in Supplementary Figure S4. As is visible in the heatmap (Supplementary Figure S4a,b, Dataset S12), T1 had a high abundance for immune system functions relative to T5 and T30. However, the microbial community in T30 was enriched in the biosynthesis of secondary metabolites. Through comparisons of the bacterial function in groups T1 and T5, we found that T30 exhibited significantly higher nitrotoluene degradation and methane metabolism (*P* < 0.05) (Supplementary Figure S4c,d, Dataset S13). Compared to T30, T5 had significantly higher degradation functions for chloroalkane and chloroalkene, as well as the metabolism of riboflavin, sphingolipid, taurine, and hypotaurine (Supplementary Figure S4d).

We allocated 1579 total fungal OTUs (Dataset S11) to fungal functional groups using the FUNGuild annotation tool. At least eight trophic modes were detected in this study, among which the saprotroph mode was the most abundant, followed by pathotroph, pathotroph-athotroph-pathogenph, pathotroph-saprotroph-symbiotroph, pathotroph-saprotroph, saprotroph-symbiotroph, and symbiotroph (Supplementary Figure S5a, Dataset S14). Sequences annotated with the pathotroph-saprotroph-symbiotroph and pathotroph-symbiotroph modes were significantly higher in the T30 group (*P* < 0.05) than those in all other groups. In contrast, the pathotroph and pathotroph-saprotroph modes were higher in the T5 group than those in other groups. At the guild level, the most abundant guilds were saprotrophs, followed by plant pathogens, soil saprotrophs, and wood saprotrophs (Supplementary Figure S5b). The relative abundances of different fungal functional guilds also differed significantly between groups. Specifically, T30 had a significantly higher abundance (*P* < 0.05) of pathogen endophyte and pathogen saprotroph and a lower abundance of wood saprotroph compared to other groups. However, T5 and T30 had significantly higher abundances of pathogen abundances (*P* < 0.05) compared to that in T1 (Supplementary Figure S5c,d, Dataset S15).

### 3.4. Abundance of Beneficial and Pathogenic Microbes and Related Gene Functions

**Figure 5:**
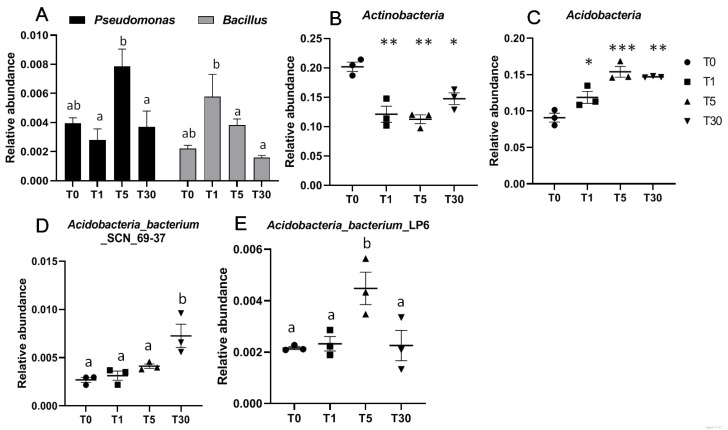
Relative abundance of bacteria in the four different groups. (**A**) Relative abundance of two beneficial bacteria, including *Pseudomonas* and *Bacillus*, were compared for the four groups; (**B**) beneficial bacterial phylum Actinobacteria; (**C**) phylum Acidobacteria; (**D**,**E**) two Acidobacteria bacteria *Acidobacteria_bacterium*_SCN_69-37 and *Acidobacteria_bacterium*_LP6. a and b represent significant differences (*p* < 0.05); Significances between different groups were compared using a one-way ANOVA test, with the results indicated on the top (*p*-value ≤ 0.05 = *, *p*-value ≤ 0.01 = **, and *p*-value ≤ 0.001 = ***).

Here, we chose the two beneficial bacteria *Pseudomonas* and *Bacillus*, which have been studied in many investigations due to their ability to improve the growth of plants [23,24]. The relative abundances of these two beneficial bacteria in the four groups displayed significant differences for T1, T5, and T30. Specifically, the abundance of *Pseudomonas* was significantly higher in T5 than that in T1 and T30, while *Bacillus* was significantly higher in T1 than that in T5 and T30 (Figure 5a, Dataset S16). Compared to the abundance of *Actinobacteria* in the bulk soil (T0), the rhizosphere soils in T1, T5, and T30 were significantly much lower (Figure 5b). In contrast, the phylum Acidobacteria abundance was significantly increased in T1, T5, and T30 compared to that in T0 (Figure 5c). In addition, the phylum Acidobacteria abundance in T5 was much higher than that in T1. In this phylum, compared to other groups, T30 was significantly enriched in *Acidobacteria bacterium* SCN_69-37 and *Cladosporium*, while T5 was significantly enriched in *Acidobacteria_bacterium*_LP6 (Figure 5d,e).

**Figure 6:**
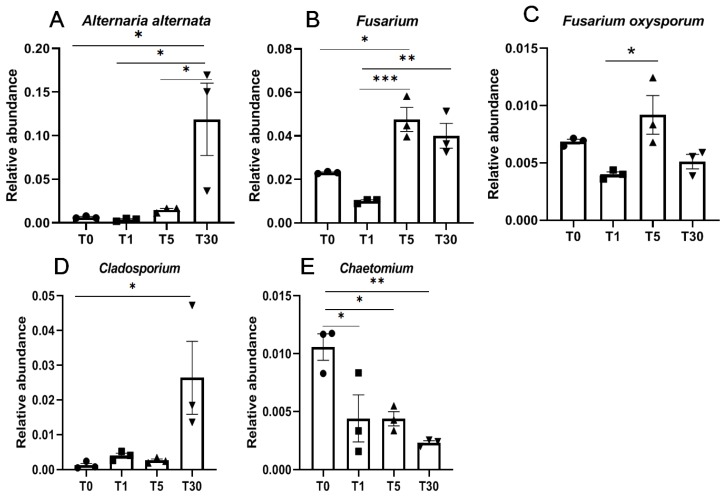
Relative abundance of beneficial and pathogenic fungi. (**A**,**C**) Pathogenic fungi species; (**B**,**D**) pathogenic fungi genera; (**E**), beneficial fungus. Significance between different groups were compared using a one-way ANOVA test, with the results indicated on the top (*p*-value ≤ 0.05 = *, *p*-value ≤ 0.01 = **, and *p*-value ≤ 0.001 = ***).

With regards to fungi, *Alternaria alternata* and *Cladosporium* were significantly enriched in T30 compared with those in other groups (Figure 6A,D, Dataset S16). Moreover, two pathogenic fungi *Fusarium* spp., including *Fusarium oxysporum*, were significantly enriched in T5 and T30, respectively (Figure 6B,C). However, the beneficial fungi *Chaetomium* was significantly lower in the rhizosphere soil of continuous sugar beet cropping compared with that in the bulk soil (Figure 6E).

**Figure 7:**
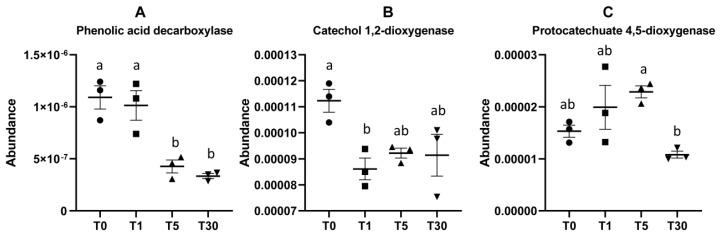
Relative abundance of enzyme-encoding genes involved in the phenolic acid degradation pathway. (**A**–**C**) Three key enzyme genes encoding phenolic acid decarboxylase, catechol 1,2-dioxygenase, and protocatechuate 4,5-dioxygenase, respectively.

The accumulation of phenolic acid in soil has been revealed to be highly correlated with consecutive years of continuous cropping [25,26]. We chose three key enzyme genes involved in the phenolic acid degradation pathway, including phenolic acid decarboxylase, catechol 1,2-dioxygenase, and protocatechuate 4,5-dioxygenase [27,28], to perform a comparative analysis of different groups. Based on the differential abundance of functional genes predicted by PICRUSt, the results showed that the enzyme genes encoding phenolic acid decarboxylase K13727 in groups T5 and T30 were significantly lower than those in T0 and T1 (Figure 7, Dataset S17). The enzyme genes encoding catechol 1,2-dioxygenase K03381 in the T0 bulk soil were significantly higher than those in other groups. The enzyme genes encoding protocatechuate 4,5-dioxygenase K04099 were detected significantly less in T30 compared with T5 (Figure 7). This indicated that the soil microbiome in the long-term mono-cropping of sugar beet would have a much lower ability to degrade the phenolic acids in soil, which could increase the pH of soil and damage the root growth.

## 4. Discussion

The continuous cropping obstacle is a common problem in the cultivation of many crops, vegetables, and medicinal herbs in China. Studies have demonstrated that the continuous cropping obstacle may be due to many factors, including soil pathogens, phytotoxic substances in root exudates, root knot nematodes, and decreases of soil nutrients [11,29]. Currently, more and more studies are focusing on detecting the roles of soil microbial communities in long-term mono-cropping practices, including black pepper, potato, and sanchi ginseng cropping systems [30,31,32,33].

Here, we conducted 16S rRNA gene and ITS gene amplicon sequencing to detect the diversity and structure of disturbance of the sugar beet rhizosphere microbiota with the increase in continuous cropping years. The most interesting finding was that the diversity of both bacterial and fungal communities decreased from T0 to T5, and then increased from T5 to T30. From the disturbance to microbial composition and structure, we observed that the species and numbers of beneficial microbes (including *Pseudomonas*, *Bacillus*, and *Actinobacteria*) decreased with the duration of mono-cropping. Pathogenic fungi, including *Fusarium*, *Alternaria*, and *Cladosporium*, significantly increased in T5 and T30. In particular, *Fusarium* and *Alternaria* are two severe plant pathogens that could lead to disease occurrence and a large-scale production reduction of sugar beet. In addition, the diversity of the pathogenic soil fungal community, including *Fusarium*, could be enhanced by root-released compounds in continuous cropping fields. We also found that the relative abundance of key enzyme genes encoding phenolic acid decarboxylase involved in the phenolic acid degradation pathway was greatly increased in T5 and T30. This indicated that the phenolic acid released by sugar beet roots [34] would be gradually enriched with consecutive years of mono-cropping, resulting in an increase of pathogenic communities and soil pH. 

In this study, the bacterial structure at the phylum and family level was not highly variable with the different years of mono-cropping, while the structure of the fungal community was changeable in different groups with distinctive dominant species. With regards to fungi, one unanticipated finding was that Ascomycota was highly abundant in T5 (50%) compared with that in other groups (lower than 43%). Mortierellomycota was highly abundant in T30 (11.6%) compared with that in other groups (lower than 8%). Pyronemataceae was the dominant family in T0 (9.8%) and T5 (14%), while Microascaceae and Pleosporaceae were the dominant families in T1 (11.5%) and T30 (12.6%), respectively. The dominant genus *Pseudombrophila* in T5 (14.3%) became one of the least abundant genera in T30 (1.5%). These observed differences in different groups further support previous reports demonstrating that the abundance and structure of fungal communities are highly influenced by continuous cropping [11,12,13,14].

Based on the functional prediction shown in the heatmap (Figure S4), we found that the occurrence of soil-borne plant pathogens increased with the duration of continuous cropping of sugar beet. Among these diseases, root rot is a severe and common disease caused by many pathogens, such as *Fusarium* spp., *Alternaria alternata*, and *Cladosporium* spp., and occurs under continuous cropping conditions [35]. As is reported, *F. oxysporum* diseases can cause significant reductions in the root yield, sugar content, and purity, and thus affect the sugar beet yield [36,37]. This pathogenic strain can remain in the soil for years, and traditional control methods are not always efficient [34]. In this study, we found that the abundance of OTU17 (*Fusarium oxysporum*) was significantly higher in the rhizosphere fungal community of continuous cropping soils than in that of bulk soil (Dataset S11), suggesting that the continuous cropping of sugar beet increased the potential pathogenic fungal density in the soil. OTU3 was identified as *Alternaria alternata*, and the abundance of this species was significantly higher in T30 and T5 than that in T1. Although there have been no reports of a sugar beet disease caused by *Alternaria alternata* in China, a previous study showed that several plants, such as onion, potato, persimmon, and pear, are hosts of this fungal genus, which could cause leaf spot, and flower bud and fruit disease [38,39,40].

A class of defensive secondary metabolites that are released by the roots of cereals, such as wheat and maize, were also found to be able to alter root-associated fungal and bacterial communities [41]. In particular, phenolic compounds were reported to be one of the most important secondary metabolites implicated in allelopathy and have been detected in both natural and managed ecosystems [21,42,43]. However, an excessive accumulation of phenolic compounds in the soil ultimately results in toxicity to the plant itself or neighbors [44]. Through detecting the functional genes involved in the phenolic acid degradation pathway in this study, we determined that the amount of phenolic acid that is released by sugar beet roots into the soil was greatly increased with the decrease of beneficial microbes due to long-term continuous cropping (Figure 7). Therefore, the interaction between phenolic acids in the rhizosphere soil and the microbial community structure may be an important cause of problems associated with the continuous cropping of sugar beet.

## 5. Conclusions

The richness and variation of the microbial population play key roles in the sustainable development of the soil quality, function, and ecosystem. This study showed significant changes in the microbial community composition at different taxa levels and the diversity of rhizosphere soil under different years of continuous sugar beet cropping. *Acidobacteria* species were significantly increased in T1, T5, and T30 compared to T0, while *Fusarium* species were highly enriched in T5, and *Alternaria* species were abundant in T30. This indicated that the pathogenic microbes would increase along with the number of years of sugar beet mono-cropping. Predictive functional analysis also indicated that soil-borne diseases would be accelerated with the increase of soil pathogenic fungi, which could seriously affect plant immunity and production. Therefore, the scientific management of sugar beet planting could be an effective measure for preventing the reproduction of plant pathogens such as *Fusarium*. Further research should be undertaken to investigate the rhizosphere soils of 10- to 20-year fields of sugar beet mono-cropping to estimate the process and mechanism underlying the adverse effects of continuous cropping.

## Supplementary Materials

The following are available online at https://www.mdpi.com/2073-4425/11/1/13/s1. Supplementary materials include one Supplementary Figure file, one Supplementary Table file, and one Supplementary Dataset file. Figure S1: Rank abundance curve of bacterial and fungal community. Figure S2: Differences of fungal abundance at dominant phylum Ascomycota between four groups with different years of continuous years. Figure S3: Differential microbial community selected via LEfSe between different groups. Figure S4: Functional prediction of bacterial community between four groups. Figure S5: Functional prediction of fungal community between four groups. Table S1: Bacterial richness and α-diversity indexes for different groups. Table S2: Fungal richness and α-diversity indexes for different groups. Supplementary Dataset: Dataset S1–Dataset S17.
