# Supplementary material for: Effects of Continuous Sugar Beet Cropping on Rhizospheric Microbial Communities
Source: Genes (Basel). 2019 Dec 22;11(1):13. doi: 10.3390/genes11010013 (PMC7017100; doi:10.3390/genes11010013)
Supplement: Supplementary file 1 [file genes-11-00013-s001.zip › Supplementary materials/Supplementary Figures.pdf]

Supplementary Figures

Fig. S1 Rank abundance curve of bacterial and fungal community. **A**, bacteria community; **B**, fungi community

Fig. S2 Differences of fungal abundance at dominant phylum Ascomycota between four groups with different years of continuous years. **A**, genus *Selinia*; **B**, genus *Pseudallescheria*; **C**, genus *Macroconia*.

Fig. S3 Comparison of bacterial and fungal abundance. **A,** bacterial abundance; **B,** fungal abundance.

Fig. S4 Functional prediction of bacterial community between four groups. **A,** functional prediction at group.cluster.level2; **B**, functional prediction at group.cluster.level3. **C**, functional differences of bacterial community between T1 and T30 using T-test ($p < 0.05$); **D**, functional differences of bacterial community between T5 and T30 using T-test ($p < 0.05$).

Fig. S5 Functional prediction of fungal community between four groups. **A**, annotation using mode; B, annotation using Guild. **C**, functional differences of fungal community between T1 and T5 using T-test ($p < 0.05$); **D**, functional differences of fungal community between T1 and T30 using T-test ($p < 0.05$).

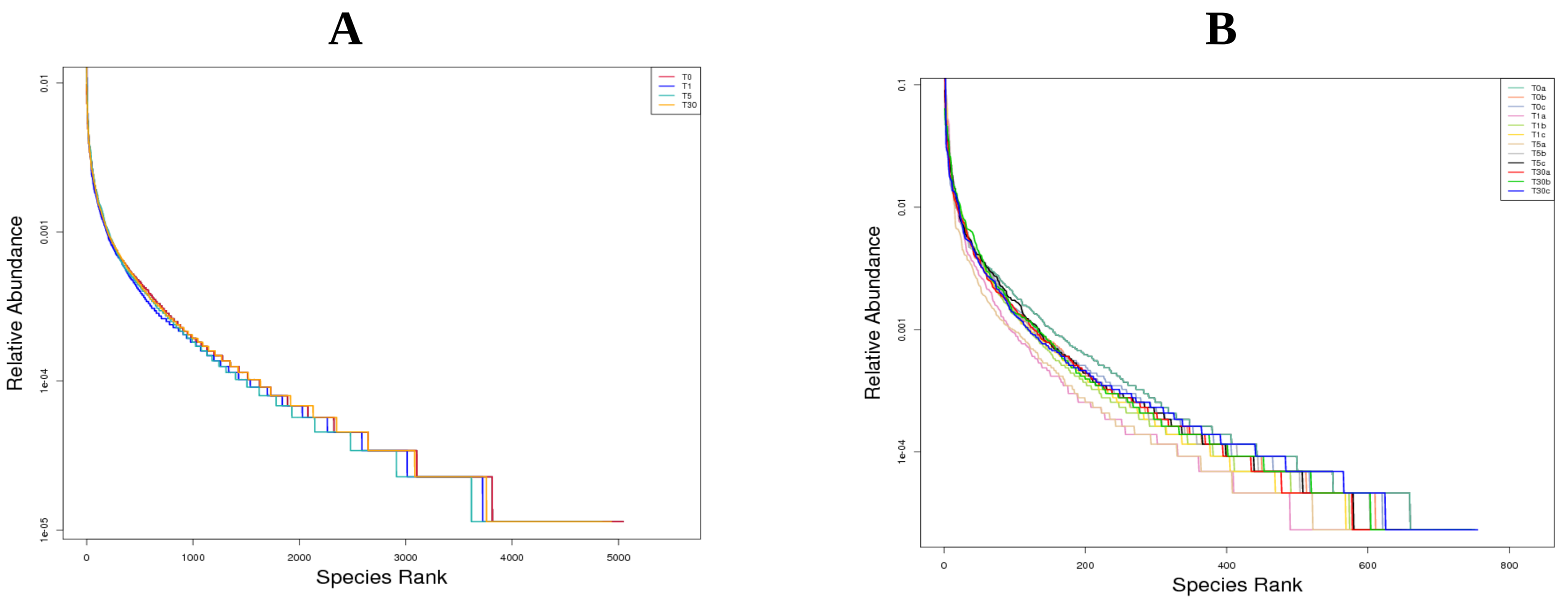

Fig. S1 Rank abundance curve of bacterial and fungal community.
A, bacteria community; **B**, fungi community

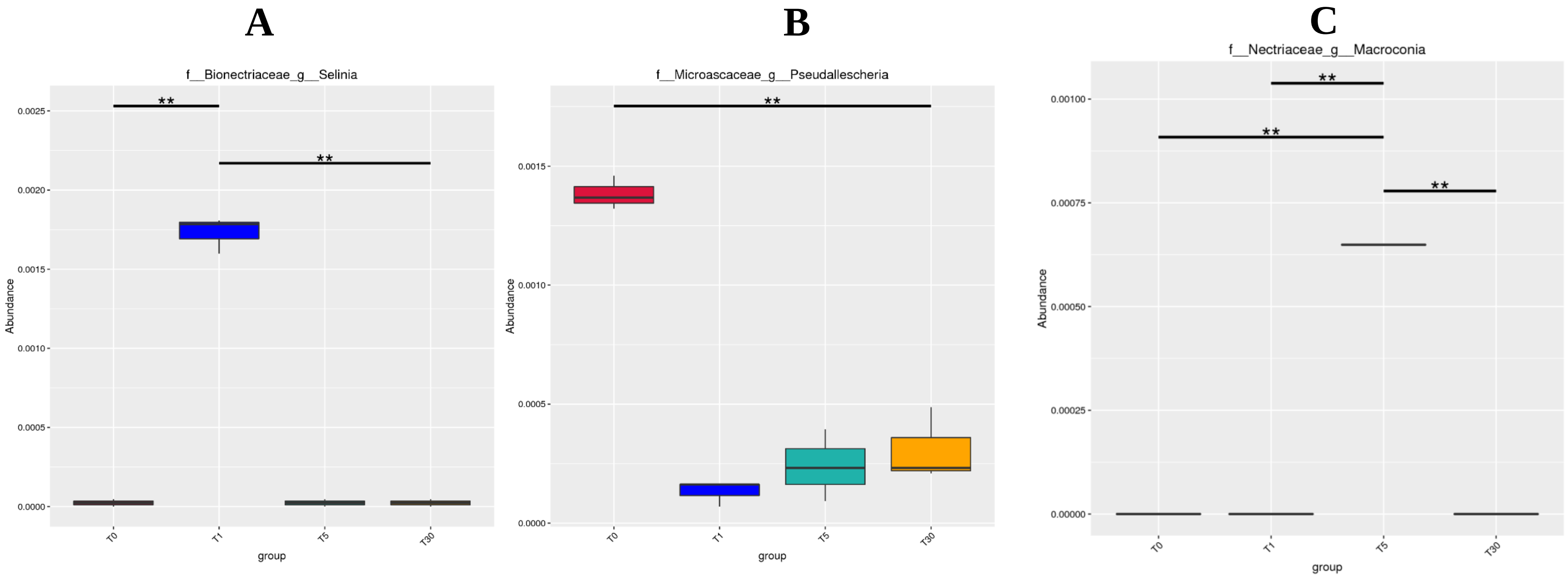

Fig. S2 Differences of fungal abundance at dominant phylum Ascomycota between four groups with different years of continuous years. A, genus *Selinia*; **B**, genus *Pseudallescheria*; **C**, genus *Macroconia*.

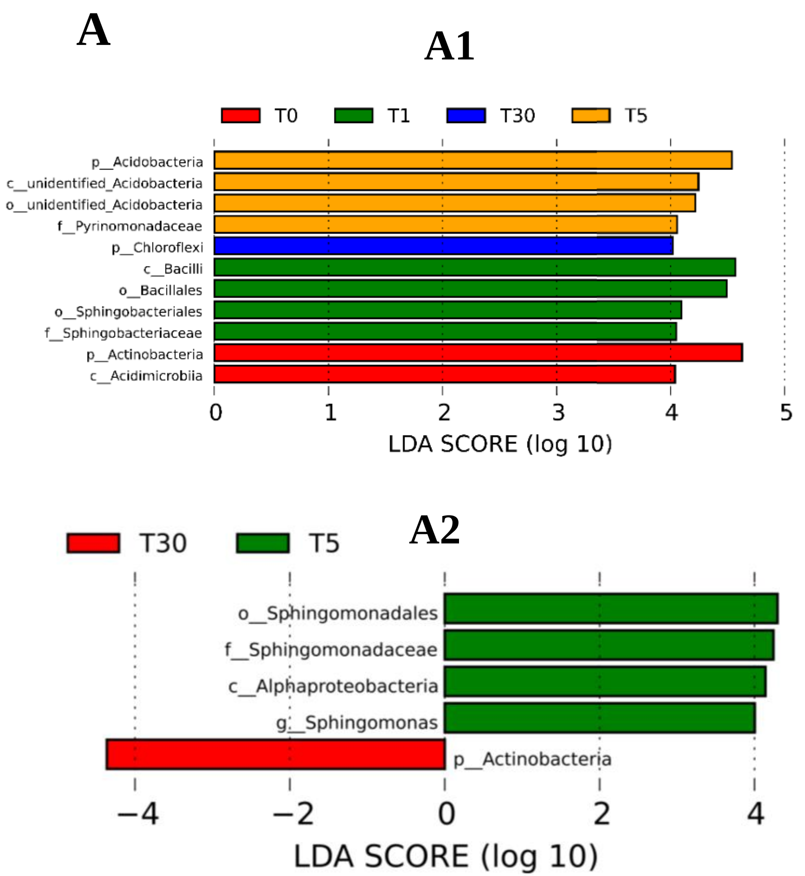

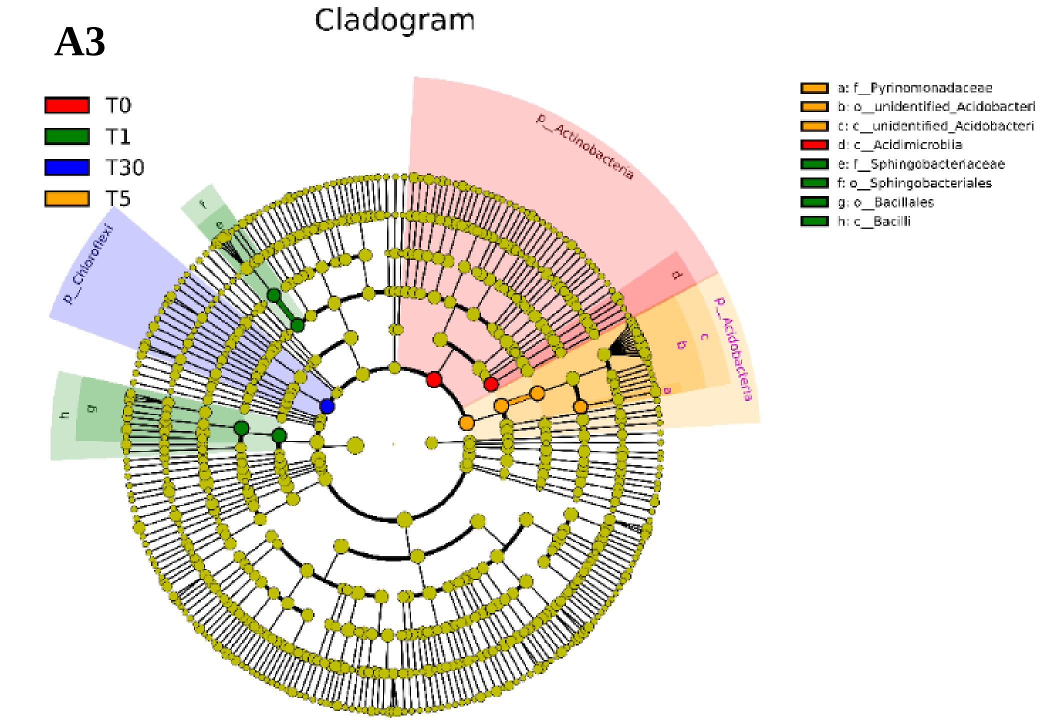

Fig. S3 Comparison of bacterial and fungal abundance. A, bacterial abundance. (**A1**, between four groups; **A2**, between T5 and T30; **A3**, cladogram cluster of four groups).

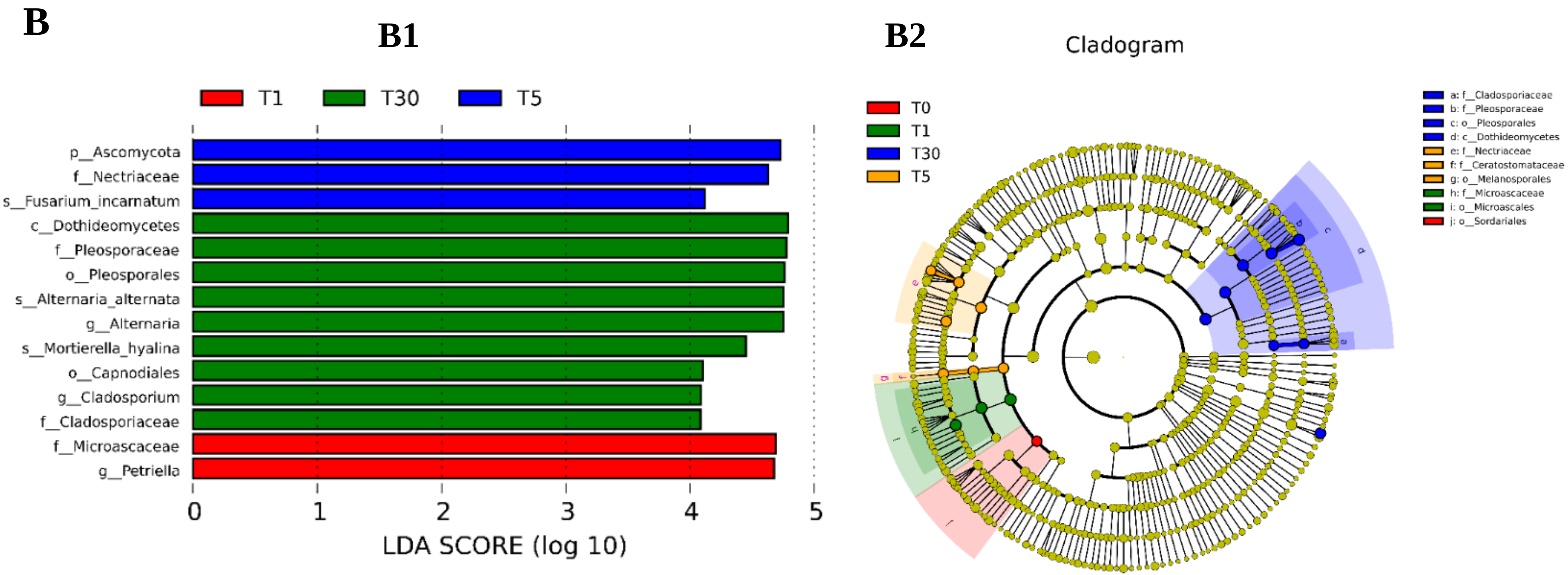

Fig. S3 Comparison of bacterial and fungal abundance. B, fungal abundance. (**B1**, between T1, T5, and T30 group; **B2**, cladogram cluster of four groups).

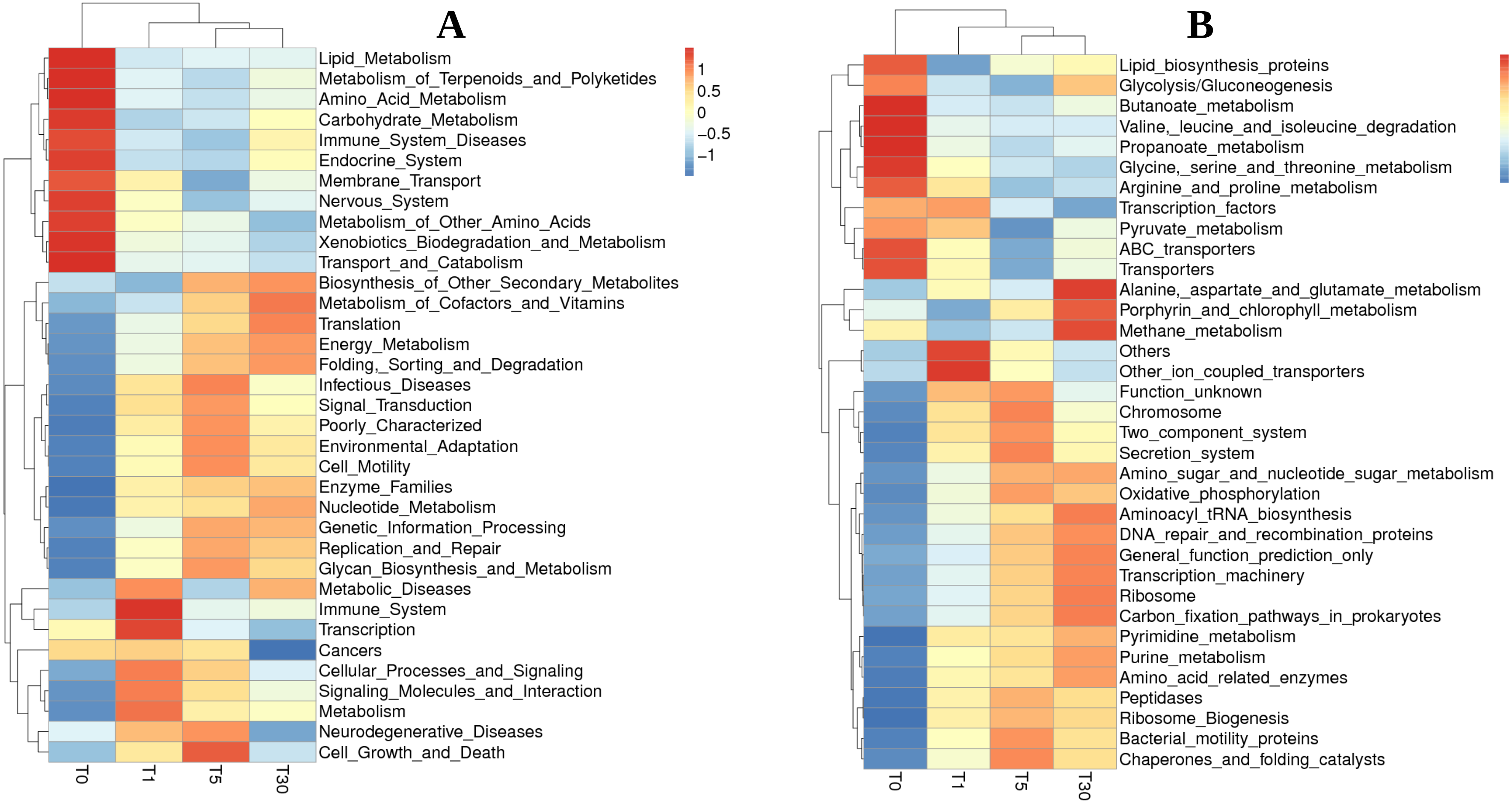

Fig. S4 Functional prediction of bacterial community between four groups. A, functional prediction at group.cluster.level2; **B**, functional prediction at group.cluster.level3.

C

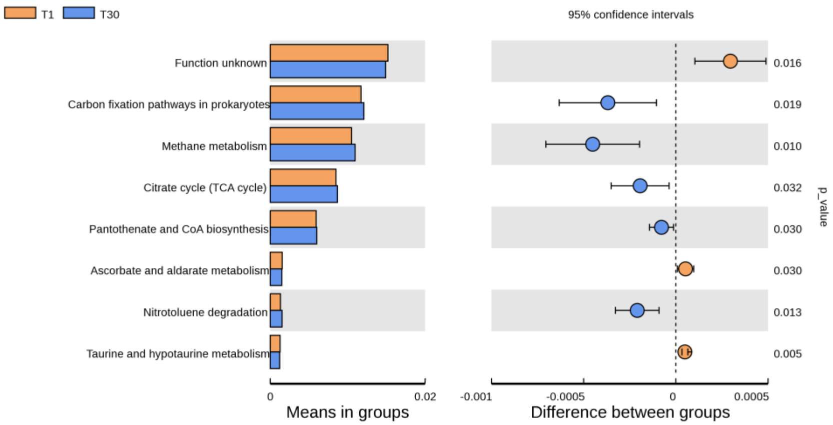

D

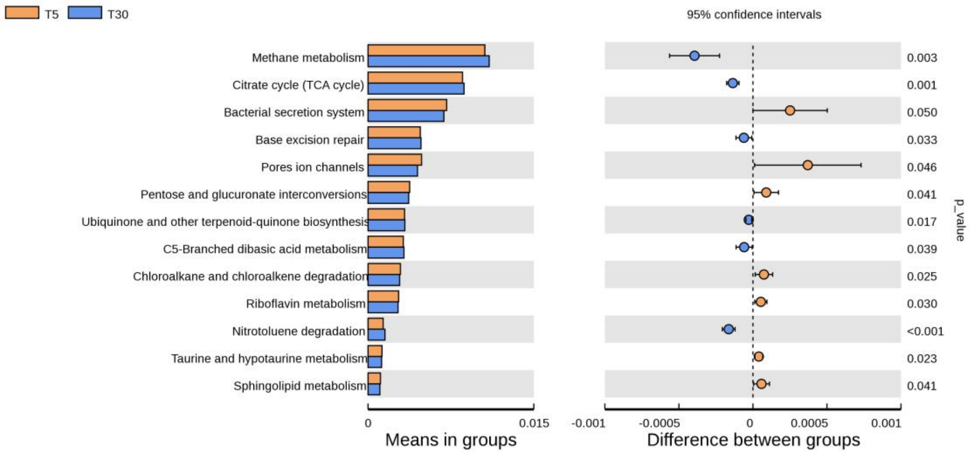

Fig. S4 (continued). C, functional differences of bacterial community between T1 and T30 using T-test ($p < 0.05$); **D**, functional differences of bacterial community between T5 and T30 using T-test ($p < 0.05$).

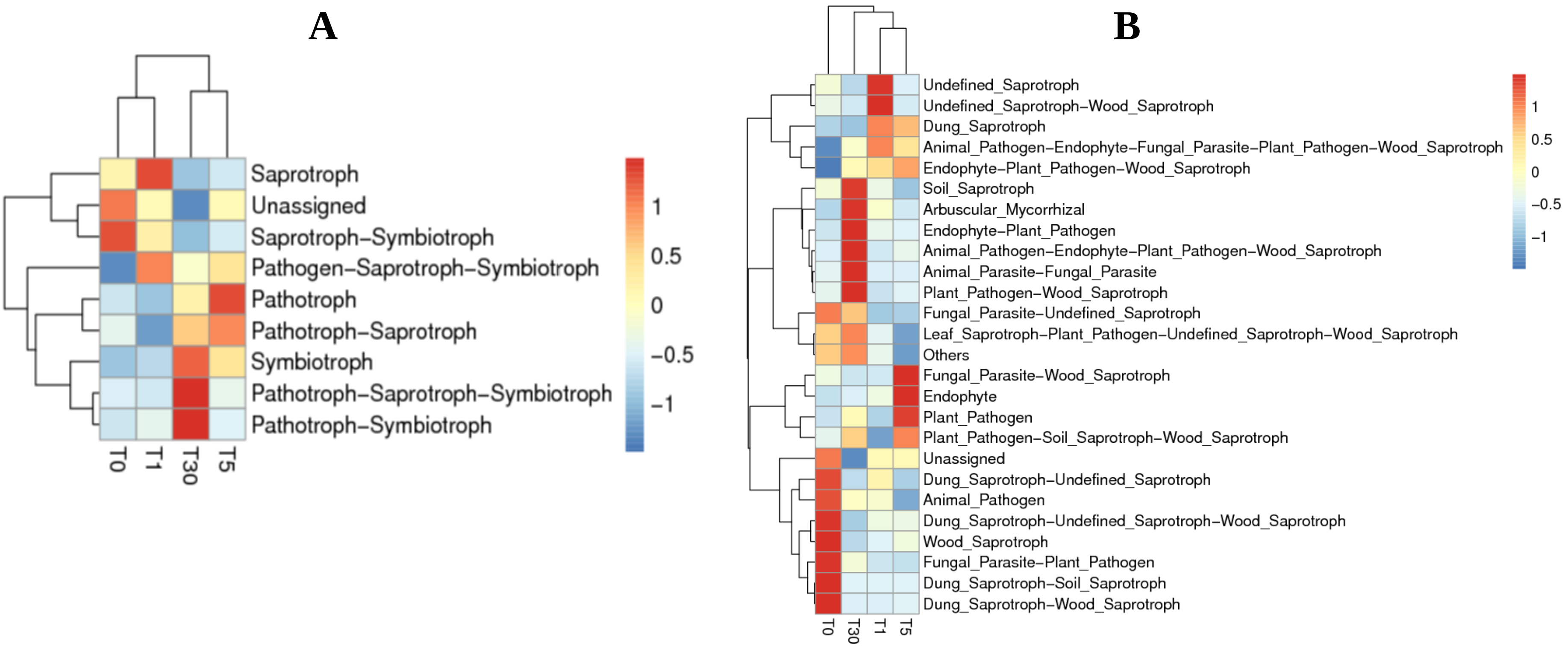

Fig. S5 Functional prediction of fungal community between four groups.

A, annotation using mode; B, annotation using Guild.

C

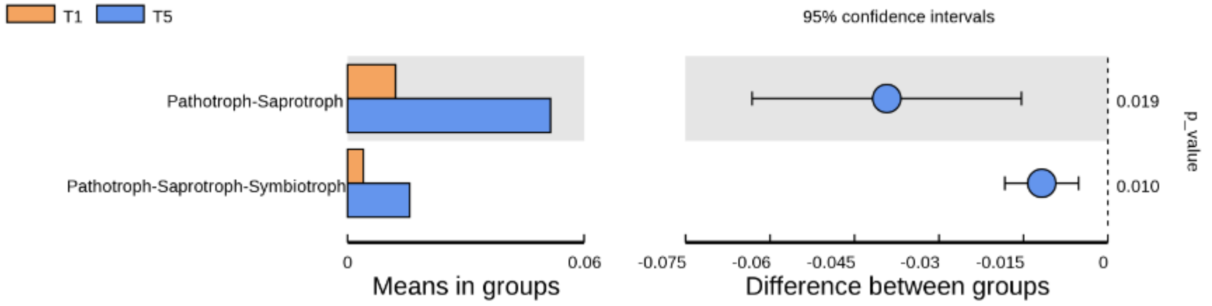

D

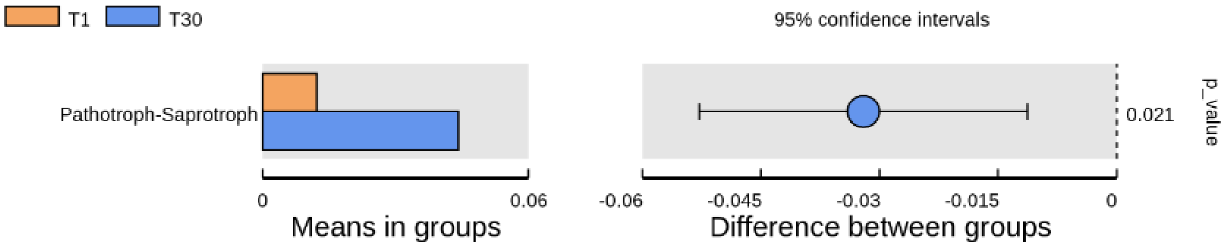

Fig. S5 (continued). C, functional differences of fungal community between T1 and T5 using T-test ($p < 0.05$); **D**, functional differences of fungal community between T1 and T30 using T-test ($p < 0.05$).
