# Supplementary material for: Effects of Continuous Sugar Beet Cropping on Rhizospheric Microbial Communities
Source: Genes (Basel). 2019 Dec 22;11(1):13. doi: 10.3390/genes11010013 (PMC7017100; doi:10.3390/genes11010013)
Supplement: Supplementary file 1 [file genes-11-00013-s001.zip › Supplementary materials/Supplementary Tables.docx]

**Supplementary Tables**

**Table S1 Bacterial richness and α-diversity indexes for different groups.**

| Group | Shannon | Observed_species | PD_whole_tree | OTU number |
| --- | --- | --- | --- | --- |
| T0 | 10.334 ± 0.085a | 4350 ± 81.505a | 288.82 ± 3.577a | 4716 ± 98a |
| T1 | 9.833 ± 0.625b | 4191 ± 32.696b | 278.169 ± 1.175b | 4577 ± 31b |
| T5 | 10.134 ± 0.076b | 4147 ± 174.949ab | 275.077 ± 7.820b | 4532 ± 182b |
| T30 | 10.246 ± 0.149ab | 4262.667 ± 126.160ab | 280.315 ± 7.431ab | 4504 ± 196b |

*Values are means ± standard deviation (n = 3), followed by the same letter for a given factor which are not significantly different (P < 0.05; Wilcoxon test).*

**Table S2 Fungal richness and α-diversity indexes for different groups.**

| Group | Shannon | Observed_species | PD_whole_tree | OTU number | OTU ratio of B/ F |
| --- | --- | --- | --- | --- | --- |
| T0 | 6.688 ± 0.250 | 727 ± 20.664 | 188.012 ± 12.531 | 798 ± 20a | 5.91 |
| T1 | 6.231 ± 0.306 | 626.667 ± 37.687 | 169.667 ± 10.406 | 702 ± 74b | 6.52 |
| T5 | 6.142 ± 0.828 | 656 ± 47.149 | 180.63 ± 11.961 | 712 ± 42b | 6.37 |
| T30 | 6.527 ± 0.209 | 720 ± 57.193 | 200.357 ± 18.623 | 791 ± 59a | 5.69 |

*Values are means ± standard deviation (n = 3), followed by the same letter for a given factor which are not significantly different (P < 0.05; Wilcoxon test). B: bacteria; F: Fungi.*
